# Optical Properties and Plasmonic Performance of Titanium Nitride

**DOI:** 10.3390/ma8063128

**Published:** 2015-05-29

**Authors:** Panos Patsalas, Nikolaos Kalfagiannis, Spyros Kassavetis

**Affiliations:** 1 Department of Physics, Aristotle University of Thessaloniki, Thessaloniki GR-54124, Greece; E-Mail: skasa@physics.auth.gr; 2 School of Science and Technology, Nottingham Trent University, Nottingham NG11 8NS, UK; E-Mail: nikolaos.kalfagiannis@ntu.ac.uk; 3 Department of Materials Science and Engineering, University of Ioannina, Ioannina GR-45110, Greece

**Keywords:** titanium nitride, optical properties, ellipsometry, plasmonics, polaritonics

## Abstract

Titanium nitride (TiN) is one of the most well-established engineering materials nowadays. TiN can overcome most of the drawbacks of palsmonic metals due to its high electron conductivity and mobility, high melting point and due to the compatibility of its growth with Complementary Metal Oxide Semiconductor (CMOS) technology. In this work, we review the dielectric function spectra of TiN and we evaluate the plasmonic performance of TiN by calculating (i) the Surface Plasmon Polariton (SPP) dispersion relations and (ii) the Localized Surface Plasmon Resonance (LSPR) band of TiN nanoparticles, and we demonstrate a significant plasmonic performance of TiN.

## 1. Introduction

Titanium nitride (TiN) is one of the most well-established engineering materials nowadays. It has been considered since the early 1970s as a superhard material for protective coatings [1,2,3,4] and, as such, has been industrially implemented. Its electronic properties were also extensively studied, as they are critical for alternative applications such as diffusion barriers in microelectronic devices [5,6,7,8,9], decorative coatings with bright golden color [10,11,12,13], ohmic contacts on III-nitride semiconductors [14,15,16,17,18,19,20,21] and Schottky contacts on Si [22,23]. Great emphasis has been given to the control of the microstructure of TiN films [24,25,26,27,28,29], which are critical for its mechanical and electronic performance. In addition to its electronic properties, TiN is appealing for electronic devices due to its compatibility with CMOS technology, because of its high electron mobility and refractory character [30,31].

An emerging field of applications for TiN is plasmonics, the science and technology of interactions of metallic nanostructures with light. In particular, the Surface Plasmon Polariton (SPP) modes on planar interfaces and the Localized Surface Plasmon Resonance (LSPR) in metallic nanoparticles are two unique phenomena that manifest exclusively at the nanoscale [32,33,34,35,36,37,38,39,40]. Plasmonics promise radical breakthroughs in electronic devices [41,42,43,44], biosensing [45,46,47,48,49], catalysis and photochemistry [50,51,52,53], solar energy harvesting [54,55,56,57,58,59,60,61], photo-detection [62,63,64], and optical storage of information [65,66,67,68,69,70], and open new pathways for the implementation of TiN in new sectors.

The most popular plasmonic metals are gold and silver. However, their melting point is low, especially when in nanoparticle form [71,72,73], making them unstable for photothermal and hot electron devices; their conduction electron mobility is very low, and, consequently, they exhibit high conduction electron losses [74,75]. TiN can overcome all these obstacles due to its high electron conductivity and mobility [14,19,30,31], high melting point and low work function, and due to the compatibility of its growth with CMOS technology, which enables its easy integration and upscaling in realistic, mainstream electronic devices.

In this work, we review the fundamental properties of TiN and its dielectric function spectra extracted from an extended literature survey of reported ellipsometry and reflectivity spectra. As a result, we critically compare the reported electronic properties (such as resistivity/mean free path, and conduction electron density—via the unscreened plasma energy). We evaluate the plasmonic performance of TiN by calculating (i) the SPP dispersion relations at planar TiN/dielectric interfaces; and (ii) the LSPR band of TiN nanoparticles based on the dielectric functions of bulk TiN. Finally, we demonstrate that the plasmonic response of nanoparticles of TiN is equivalent, if not superior, to that of nanoparticles of noble metals.

## 2. Results and Discussion

### 2.1. Fundamental Features and Methods of Growth

TiN belongs to the transition metals of the group IVb of the periodic table of elements. As such, it has four valence electrons and their configuration is 3*d^2^*4*s^2^*. Ti forms bonds with N atoms (valence electronic configuration 2s^2^2p^3^). While various nitride phases such as the Ti_2_N [76,77] and Ti_3_N_4_ [78,79] were reported, the most stable and durable nitrides of these metals are those in the cubic rocksalt B1-TiN structure, also called δ-TiN. The B1-TiN is characterized by gold-like yellow color [10,11,12,13], high hardness [1,2,3,4], electrical conductivity [15,19], and refractory character. Its electronic conductivity is due to the partially filled valence Ti-3*d* orbitals that are not completely hybridized with the N-2*p* electrons [80,81,82].

A wide variety of fabrication techniques have been used for the growth of B1-TiN films, such as Magnetron Sputtering (MS) [83,84,85,86,87,88,89,90], Cathodic Vacuum Arc (CVA) [91,92,93], Chemical Vapor Deposition (CVD) [94,95], Atomic Layer Deposition (ALD) [96,97], Pulsed Laser Deposition [15,16,98,99], Ion Beam Assisted Deposition (IBAD) [100] and High Power Impulse Magnetron Sputtering (HIPIMS) [101]. The numerous works on the growth of B1-TiN led to an unprecedented understanding and control of its microstructural features, such as the grain size, orientation, columnar or globular type of growth, *etc.* [102,103,104,105,106,107].

### 2.2. Optical Properties of TiN Films

The optical properties of B1-TiN, which are relevant to plasmonic applications, have been also a subject of intense experimental research using mostly spectroscopic ellipsometry (SE) in all of its varieties, and spectral reflectivity measurements usually at normal incidence [14,15,16,18,19,82,108,109,110,111,112,113,114,115,116,117,118,119,120,121,122,123,124,125,126,127,128,129,130,131,132,133]. SE measures the ellispometric angles Ψ-Δ, which are associated with the ratio ρ_f_ of the Fresnel reflection coefficients for *s*- and *p*-polarization [134]:

(1) $$\rho_{f}=tan\psi ∙e^{i\Delta }$$

Then, the real and imaginary parts of the dielectric function $\tilde{\varepsilon }=\varepsilon_{1}+i\varepsilon_{2}$ can be analytically calculated from ρ*_f_* and the angle of incidence ϕ(usually in the range 55°–75°) [134]:

(2) $$\tilde{\varepsilon }=sin^{2}\text{ϕ}∙\left[1+tan^{2}\varphi {\left(\frac{1-\rho_{f}}{1+\rho_{f}}\right)}^{2}\right]$$

On the other hand, the real and imaginary parts of the complex refractive index $\tilde{n}=n+ik$ can be extracted from the spectral reflectivity measurements at normal incidence *R* via the relations [135]:

(3) $$n=\frac{1-R}{1+R-2cos\theta \sqrt{R}},k=\frac{-2sin\theta \sqrt{R}}{1+R-2cos\theta \sqrt{R}}$$

where θ is the phase change due to reflectivity, which is determined by Kramers-Kronig integration [135]:

(4) $$\text{θ}\left(\omega_{0}\right)=\frac{1}{\pi }\overset{\infty }{\underset{0}{\int }}ln\left|\frac{\omega +\omega_{0}}{\omega -\omega_{0}}\right|\frac{dln\sqrt{R\left(\text{ω}\right)}}{d\omega }d\text{ω}$$

Alternatively, the *n, k* can be also determined by fitting the spectral reflectivity curves using specific dispersion relation models, as we will discuss below. The complex dielectric function and the complex refractive index are equivalent and interchangeable, as they are analytically correlated:

(5) $${\text{ε}}_{1}=n^{2}-k^{2},\;{\text{ε}}_{2}=2nk$$

In the case of a thin film grown on a bulk substrate, the measured spectra by SE or spectral reflectivity accounts the effect of the substrate and film’s thickness, in addition to the film’s optical properties. Opaque TiN films should be thicker than 100 nm in order to measure directly their complex dielectric function, without any contribution from the substrate; else, a three-phase model (air/homogeneous film/semi-infinite substrate) [136] is required to determine the complex dielectric function.

**Figure 1:**
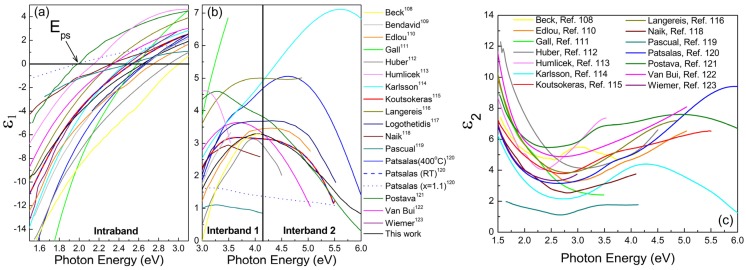
(**a**), (**b**) ε_1_, and (**c**) ε_2_ spectra of TiN reported by several groups worldwide.

In this work, we retrieved and critically review the optical data of TiN from [108,109,110,111,112,113,114,115,116,117,118,119,120,121,122,123,124,125,126,127,128]. Table 1 lists the samples grown and the spectra measured by numerous groups worldwide regarding the optical properties of TiN. It includes information relevant to the growth conditions, morphology, and optical measurement conditions, as well as the corresponding references [108,109,110,111,112,113,114,115,116,117,118,119,120,121,122,123,124,125,126,127,128]. In order to have a unified picture and compare all the available data in the literature, when the *n*, *k* values are reported in the original references, we calculated the ε_1_, ε_2_ values using Equation (5). The ε_1_, ε_2_ from [108,109,110,111,112,113,114,115,116,117,118,119,120,121,122,123] are presented in Figure 1. There is a substantial variation and scattering of the reported experimental spectra due to the sensitivity of TiN’s optical performance to various factors, such as stoichiometry ([N]/[Ti] ratio), impurities (residual oxygen in sputter growth or chlorine in CVD growth, post growth oxidation), grain size (which affects the mean free path of the conduction electrons, as we will discuss in more detail below) and density/porosity (which affect the conduction electron density [120]).

The ε_1_ spectra have attracted particular attention, and are considered to be characteristic of the TiN’s chemical and structural quality [117]. The first spectral region (up to 3 eV) is characterized by negative values of ε_1_ due to the interaction of light with the conduction electrons of TiN, *i.e.*, due to intraband absorption. In particular, and of major importance, is the screened plasma energy *E*_ps_ = *ħ*ω_ps_, which is the spectral energy at which TiN’s ε_1_ = 0. *E*_ps_ may be affected by both the intraband and interband characteristics and ranges from 2 to 2.95 eV. E_ps_ was reported to be an indicator of the stoichiometry of TiN_x_, and gets the value 2.65 eV for *x* = 1 [117]; despite of the efficiency of this phenomenological observation, *E*_ps_ does not have any other solid physical meaning but it is critical for the plasmonic behavior of TiN-based SPP devices, as we will show below.

**Table 1:** The titanium nitride (TiN) spectra reviewed in this work.

No	Growth Technique	Substrate	Thickness (nm)	Morphology	Measurement Technique ^#^	Instrument *	Analysis Model	Reference	Comments
1	N/A	Stainless Steel	Opaque	N/A	SE	RPE by SOPRA	N/A	[108]	Stoichiometry determined by Auger Electron Spectroscopy (AES)
2	FCVA	Si(100)	150	N/A	SE	RAE	D	[109]	Stoichiometry x = 0.97 determined by Rutherford Backscattering (RBS)
3	Magnetron Sputtering	Fused Silica	Opaque	N/A	VASE	N/A	D+1L	[110]	Stoichiometry determined by RBS
4	UHV Sputtering	MgO(100)	200	Epitaxial	ORS	Perkin Elmer	D+2L	[111]	(100) Oriented identified by X-Ray Diffrcation (XRD); composition determined by RBS and X-Ray Photoelectron Spectroscopy (XPS); ORS measure-ments acquired using an integrating sphere; ORS normalization was performed via a Si(100) wafer
5	Me-PIII	Si(100)	100–500	Polycrystalline	SE	RAE	D+iL	[112]	Stoichiometry x = 0.95 determined by Elastic Recoil Detection Analysis (ERDA); crystal structure identified by XRD
6	IBAD	Si(100)	50–100	Polycrystalline	VASE	RAE	D+1L	[113]	Stoichiometry determined by XPS and RBS; crystal structure identified by XRD; existent O impurities
7	CVD	Polished Mo	~1000	Polycrystalline	ORS	Perkin Elmer	D+1L	[114]	Used TiCl_4_, H_2_, and N_2_ precursors; stoichiometry close to 1 determined by Electron Probe Microanalysis (EPMA) and by the cubic lattice size (0.424 nm) determined by XRD; no Cl impurities; C impurities <0.2% wt; Normalization of the spectra was performed via an evaporated Al mirror; Reflectivity spectra combined with Kramers-Kronig analysis to extract ε_1_, ε_2_
8	DIBS	Si(100)	>300	Globular polycrystalline	VASE	RPE by SOPRA	D+2L	[115]	Stoichiometry x = 1 determined by AES and XPS; crystal structure identified by XRD
9	ALD	H-terminated Si	>100	Polycrystalline	*in situ* SE	RCE by J.A. Woolam	D+2L	[116]	Used TiCl_4_, H_2_, and N_2_ precursors; x < 1
10	Magnetron Sputtering	Ti/Si(100)	70	Columnar Polycrystalline	*in situ* SE	PME by JY-Horiba	N/A	[117]	Microstructure identified by Transmission Electron Microscopy; no information on stoichiometry
11	Magnetron Sputtering	c-Sapphire	30	Epitaxial	VASE	RCE by J.A. Woolam	D+1L	[118]	The TiN[111]/Al_2_O_3_[0006] epitaxy was confirmed by XRD
12	PECVD	Glass	Opaque	N/A	SE	RAE	N/A	[119]	Used TiCl_4_, H_2_, and N_2_ precursors; no information on composition and structure
13	Magnetron Sputtering	Si(100)	>100	Polycrystalline	*in situ* SE	PME by JY-Horiba	D+2L	[120]	Growth at 400 °C, V_b_=-100 V; Stoichiometry close to 1 confirmed by XPS; crystal structure and (100) texture identified by XRD
14	Magnetron Sputtering	Si(100)	>100	Polycrystalline	*in situ* SE	PME by JY-Horiba	D+2L	[120]	Growth at RT and Bias voltage of -120 V; Stoichiometry x = 1 determined by XPS; crystal structure and (100) texture identified by XRD
15	Magnetron Sputtering	Si(100)	>100	Columnar Polycrystalline	*in situ* SE	PME by JY-Horiba	D+2L	[120]	Growth at RT and V_b_ = −20 V; Stoichiometry x = 1.12 determined by XPS; crystal structure and (111) texture identified by XRD
16	Sputtering	Thick thermal SiO_2_	107.1	N/A	ORS	Shimadzu	D+2L	[121]	Spectral normalization was performed via a Si wafer
17	ALD	100 nm thermal SiO_2_	10	N/A	*in situ* SE	RCE by J.A. Woolam	D+2L	[122]	Used TiCl_4_ and NH_3_ precursors; Overstoichiometry determined by XPS; Existent Cl-impurities
18	Magnetron Sputtering	Si(100)	400	Polycrystalline	SE	PME by JY-Horiba	D	[123]	Stoichiometry 1.03 > x > 1 determined by ERDA; crystal structure identified by XRD
19	PLD	Si(100)	>100	Polycrystalline	VASE	PME by JY-Horiba	D+2L	This work	Growth at Room Temperature; Stoichiometry x = 1 determined by *in situ* XPS; crystal structure and (111) texture identified by XRD
20	Magnetron Sputtering	Glass	N/A	N/A	SE	Null Ellips.	N/A	[124]	Measurement acquired at 70° angle of incidence and in p-polarization
21	N/A	N/A	N/A	N/A	ORS	N/A	N/A	[125]	Reflectivity confirmed by *ab initio* calculations for stoichiometric TiN
22	Plasma-assisted Evaporation	Si or Glass	85	Polycrystalline	*in situ* SE	N/A	N/A	[126]	Reduced reflectivity values due to short thickness (films not completely opaque)
23	Magnetron Sputtering	Ck35 Carbon steel	N/A	Columnar Polycrystalline	SE	RAE	N/A	[127]	Stoichiometry determined by EPMA; crystal structure identified by XRD
24	Magnetron Sputtering	Stainless steel	>2000	Polycrystalline	ORS	Beckman	D	[128]	Stoichiometry determined by EPMA; crystal structure identified by XRD

^#^ SE = Spectroscopic Ellipsometry, VASE = Variable-Angle Spectroscopic Ellipsometry, ORS = Normal-Incidence Optical Reflectivity Spectroscopy unless otherwise specified; * RAE = Rotating Analyzer Ellipsometer, RCE = Rotating Compensator Ellipsometer, RPE = Rotating Polarizer Ellipsometer, PME = Phase-Modulated Ellipsometer.

**Figure 2:**
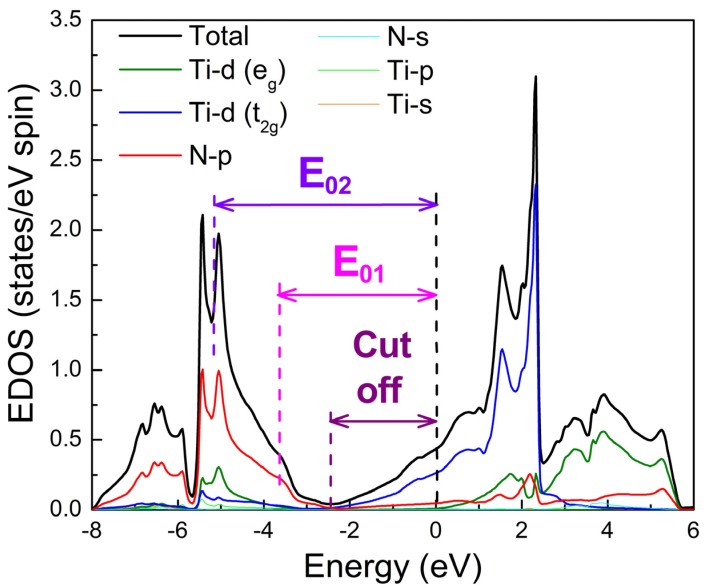
Electron density of states for TiN. Zero energy stands for the Fermi level and is denoted by a vertical, dashed, black line. The vertical dashed purple, magenta, and blue lines denote the cut off energy, and the energy positions of the two interband transitions *E*_01_, *E*_02_, respectively.

Beyond 3 eV, we observe positive values of ε_1_, which are associated with the interband absorption; these bands are also observed in the ε_2_ spectra. The interband absorption, which has the form of two distinct peaks, would be solidly identified, if we considered the electron density of states (EDOS) of TiN. Figure 2 presents EDOS calculations for TiN from [82], which are based on the linear augmented plane wave (LAPW) method within the density functional theory using the Wien*2k* software [137] and using the Generalized Gradient Approximation (GGA) in the form given by Perdew-Burke-Ernzerhof (PBE96) [138].

**Figure 3:**
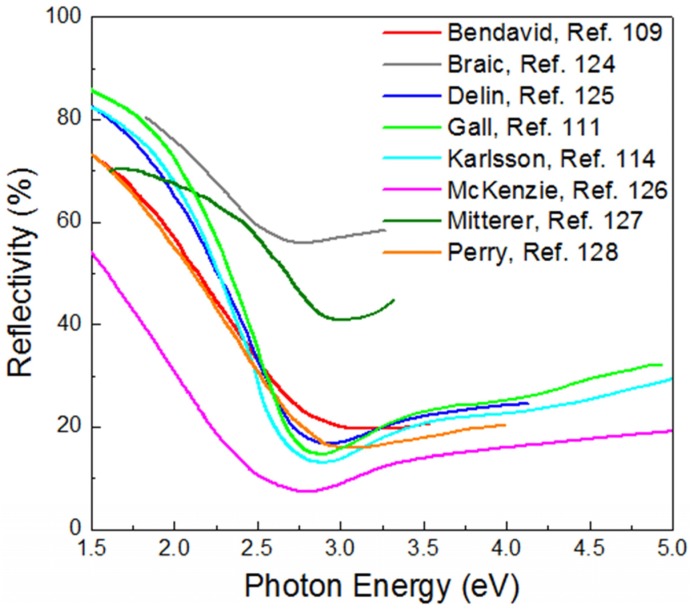
Optical reflectivity spectra of TiN at normal incidence reported by several groups. The absolute values vary due to TiN film’s thickness or normalization standards; however, they all share the same qualitative features. In particular, above 2.5 eV all spectra exhibit reduced reflectivity values due to dielectric losses.

The observed wide interband absorption originates from the states located 2.5–5.5 eV below the Fermi level, which are predominantly due to N-*p* electrons. A N*-p→*Ti*-d(t_2g_)* transition is in accordance with the selection rules for photonic excitation (*Δl* = 0, ±1) and the spectral separation between the N-*p* states and the Fermi level that intercept the *t_2g_* conduction band of the metals. The cut off energy (the maximum energy below the Fermi level, where the partial EDOS of the N-*p* states gets non-zero values) is around 2.5 eV and defines the threshold at which the dielectric losses contribute to the optical repsonse of TiN. These dielectric losses have a severe impact on the optical reflectivity spectra of TiN at normal incidence, as shown in Figure 3.

At around 3.5 eV below the Fermi level, there is the local minimum *E*_1_ in the N-*p* band; this is in excellent agreement with most of the experimental obsrevations presented in Figure 1b,c. The global maximum of ε_2_, which is defined as the spectral separation between the EDOS maximum of the N-*p* and the Fermi level defines the second absorption band E_2_, which is manifested over 5 eV.

The dielectric function of TiN films can be analyzed, via appropriate modeling in order to take into account contributions of intraband and interband transitions as described by a Drude term and several Lorentz oscillators, respectively [120,139]:

(6) $$\tilde{\text{ε}}(\text{ω})={\text{ε}}_{\infty }-\frac{{\text{ω}}_{\text{pu}}^{2}}{{\text{ω}}^{2}-i{\text{Γ}}_{\text{D}}\text{ω}}+\sum_{j=1}^{m}\frac{f_{\text{j}}\cdot {\text{ω}}_{\text{oj}}^{2}}{{\text{ω}}_{\text{oj}}^{2}-{\text{ω}}^{2}+i\gamma_{\text{j}}\text{ω}}$$

In Equation (2) ε_∞_ is a background constant, larger than unity, which is due to higher-energy contributions (beyond the experimental spectral range) referring to transitions that are not taken into account by the Lorentz term(s). Each of the Lorentz oscillators is located at an energy position *E*_oj_ *= ħ*w_oj_, with strength *f*_j_ and damping (broadening) factor γ_j_.

The Drude term is characterized by the unscreened plasma energy $E_{\text{pu}}=\hbar {\text{ω}}_{\text{pu}}$ and the damping factor Γ_D_ *E*_pu_ depends on the concentration of the conduction electrons in the film and is defined by the relation [120,139]:

(7) $$E_{\text{pu}}=\hbar {\text{ω}}_{\text{pu}},\;{\text{ω}}_{\text{pu}}=\sqrt{\frac{\;N\;e^{2}}{{\text{ε}}_{o}m^{*}}}$$

where N is the conduction electron density, e is the electron charge, ε_o_ is the permittivity of free space and m^*^ is the electron effective mass, in SI units. Since *E*_pu_ is directly correlated with the conduction electron density, it can be used to quantify the metallic character of the TiN. Γ_D_ is due to the scattering of electrons and according to the free-electron theory it is related to the electron relaxation time through the relation [120,139]:

(8) $${\text{τ}}_{\text{D}}=\frac{\hbar }{\Gamma_{\text{D}}(eV)}$$

The ω_pu_ and τ_D_ are also closely related with the electrical resistivity (ρ). The relaxation time of conduction electrons is associated with the film resistivity through the relation [120,140]:

(9) $${\text{τ}}_{\text{D}}=\frac{m^{*}}{\text{ρ }N\;e^{2}}\text{ or ρ}=\left(\frac{4\text{π}}{\hbar }\right)\cdot \frac{\Gamma_{\text{D}}}{{\text{ω}}_{\text{pu}}^{2}}$$

Changes of ω_pu_ may be attributed, according to Equation (7), either to changes in free electron density or to the electron effective mass [19]. In any case, the increase of ω_pu_ is strongly correlated with the enhancement of TiN metallic character expressed in terms of electrical resistivity. 

Based on the above, the relaxation time can be used to calculate the mean free path (MFP) of the conduction electrons in TiN. The MFP for nanocrystalline TiN or for TiN nanostructures and nanoparticles is mostly due to scattering of electrons at the particle surfaces. MFP can be expressed, in terms of the relaxation time and the velocity (v_F_) at the Fermi surface [141]:

(10) $$\frac{1}{{\text{τ}}_{\text{D}}}=\frac{1}{{\text{τ}}_{\text{bulk}}}+\frac{v_{\text{F}}}{\text{MFP}}\Leftrightarrow \Gamma_{\text{D}}=\Gamma_{\text{bulk}}+\frac{v_{\text{F}}}{\text{MFP}}\Leftrightarrow \text{MFP}=\frac{v_{\text{F}}}{\Gamma_{\text{D}}-\Gamma_{\text{bulk}}}$$

where τ_bulk_ is the electron relaxation time of the bulk TiN. The velocity at the Fermi surface is calculated according to the free electron model through the relation [120,140]:

(11) $$v_{\text{F}}=\left(\frac{\hbar }{m^{*}}\right)\cdot {\left(3{\text{π}}^{2}\;N\right)}^{1/3}=\hbar {\left(\frac{0.75\text{π}}{{\left(m^{*}\;e\right)}^{2}}\right)}^{1/3}\cdot {\text{ω}}_{\text{pu}}^{2/3}$$

The velocity of thermal movement of electrons (*v*_T_ = (k_B_T/2m^*^)^1/2^ = 4.77 × 10^6^ cm/s) is smaller by two orders of magnitude than v_F_ and thus it can be reasonably neglected in the calculation of MFP:

(12) $$\text{MFP}={\left(\frac{0.75\text{π}}{{\left(m^{*}\;e\right)}^{2}}\right)}^{1/3}\cdot \frac{\hbar^{2}{\text{ω}}_{\text{pu}}^{2/3}}{\Gamma_{\text{D}}-\Gamma_{\text{bulk}}}$$

The Γ_bulk_ for TiN was determined to be 0.13 eV by *ab initio* calculations [82]; note that in [120], the MFP was determined using the crude assumption Γ_bulk_ = 0 eV, because at that time there were no relevant data available for Γ_bulk_, therefore, the reported values in Table 2 are more realistic approximations of the real conduction electron MFP for TiN.

In this work we fitted most of the spectra presented in Figure 1 by Equation (6) using the Drude term and two Lorentz oscillators (m = 2), which simulate the contributions of the two interband transitions, *E*_01_, *E*_02_ defined in Figure 2, in order to provide a quantitative comparison; we call this model D2L. Spectra of non identified thickness or reference optical data of the substrate are excluded from the comparison.

**Figure 4:**
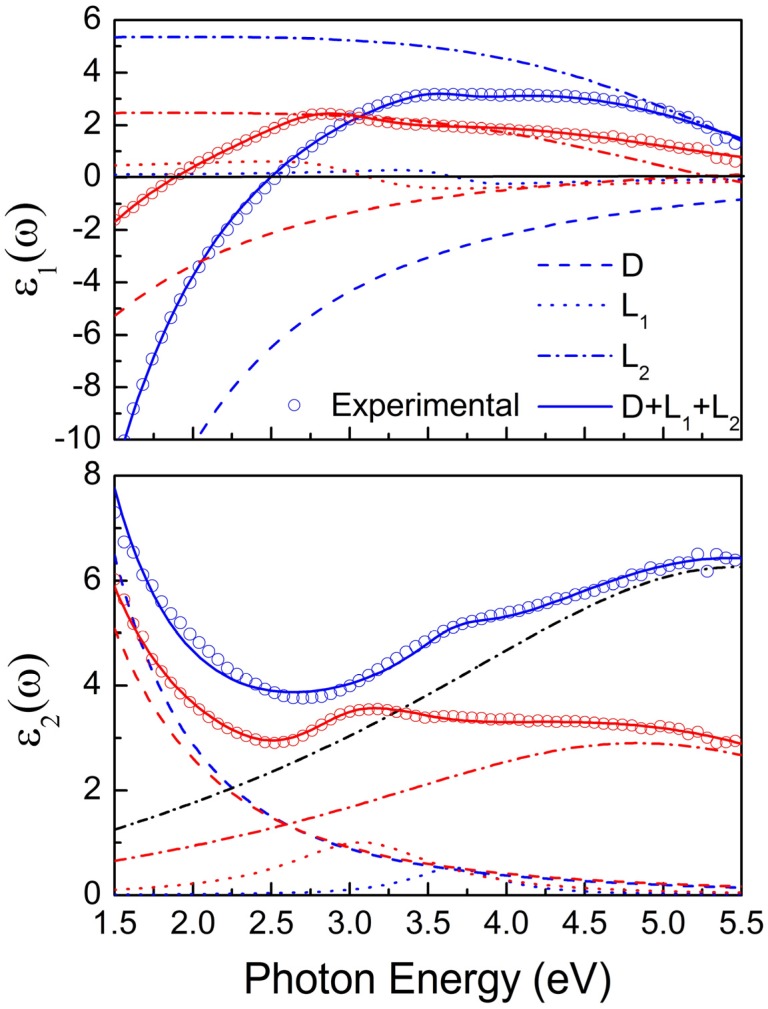
The experimental (open circles) and the fitted (solid lines) real (ε_1_) and the imaginary (ε_2_) parts of the dielectric function of two representative TiN films, 100 nm thick, deposited at RT and V_b_ = −40 V (red points and lines), (b) V_b_ = −120 V (blue points and lines). The individual contributions of the Drude (D) term (dashed lines) and the two Lorentz oscillators (L_1_, L_2_, dotted, and dashed-dotted lines) are also shown. The experimental data are taken from [120].

Representative experimental dielectric function spectra along with the corresponding D2L fit results and the convolution to the individual contributions of the Drude term and the two Lorentz oscillators for two TiN films exhibiting high (blue symbols and lines) and low (red symbols and lines) E_ps_ values are presented in Figure 4. The results of the fits, as well as the values of resistivity and MFP [according to Equations (9) and (12)], are presented in Table 2. Note that the presented resistivity values are consistent with the reported resistivity acquired by electrical measurements [120,142,143,144,145].

**Table 2:** The results of the Drude-Lorentz fits on the complex dielectric function spectra of TiN reported by various groups.

Reference	Intraband		Interband 1			Interband 2			Composite		Properties	
	*E*_pu_ (eV)	Γ_d_ (eV)	*f*_1_	*E*_01_ (eV)	γ_1_ (eV)	*f*_2_	*E*_02_ (eV)	γ_2_ (eV)	*E*_ps_ (eV)	ε_∞_	MFP (nm)	ρ(μΩ cm)
Edlou *et al.* [110]	7.248	0.639	0.232	2.066	0.544	4.039	5.645	3.621	2.75	2.352	19.9	94
Gall *et al.* [111]	9.766	0.349	0.395	2.195	0.816	5.278	8.228	4.041	2.59	8.784	56.5	28
Huber *et al.* [112]	8.080	0.860	3.536	5.139	3.043	N/A	N/A	N/A	2.90	2.998	14.9	102
Humlicek *et al.* [113]	6.421	0.859	3.118	3.765	1.547	N/A	N/A	N/A	2.15	3.839	12.8	161
Koutsokeras *et al.* [115]	7.051	0.629	0.383	3.713	1.333	4.349	5.878	4.282	2.55	2.129	20.0	98
Langereis *et al.* [116]	7.210	0.806	2.685	4.480	2.644	1.274	5.355	0.879	2.32	3.871	15.0	120
Naik *et al.* [118]	5.707	0.165	0.195	2.264	0.722	2.312	4.894	3.875	1.99	2.667	247.3	39
Patsalas *et al.* [12] 400 °C, V_b_ = −100 V	7.750	0.350	0.100	3.682	0.712	5.620	6.260	3.875	2.67	1.850	48.2	45
Patsalas *et al.* [120] RT, V_b_ = −120 V	6.926	0.589	0.183	3.690	0.943	4.877	5.967	4.882	2.65	1.872	21.5	95
Patsalas *et al.* [120] RT, V_b_ = −40 V, x = 1.1	4.493	1.379	0.550	3.761	2.075	1.639	6.674	5.553	1.98	1.198	5.9	527
Postava *et al.* [121]	6.357	0.738	0.146	3.478	0.760	7.008	5.789	5.933	1.99	1.957	15.3	141
Wiemer *et al.* [123]	7.067	0.438	4.218	5.084	3.349	N/A	N/A	N/A	2.48	3.236	32.4	68
TiN by PLD, this work	6.944	0.726	3.962	5.521	4.117	1.041	7.823	2.199	2.64	1.198	16.5	116

**Figure 5:**
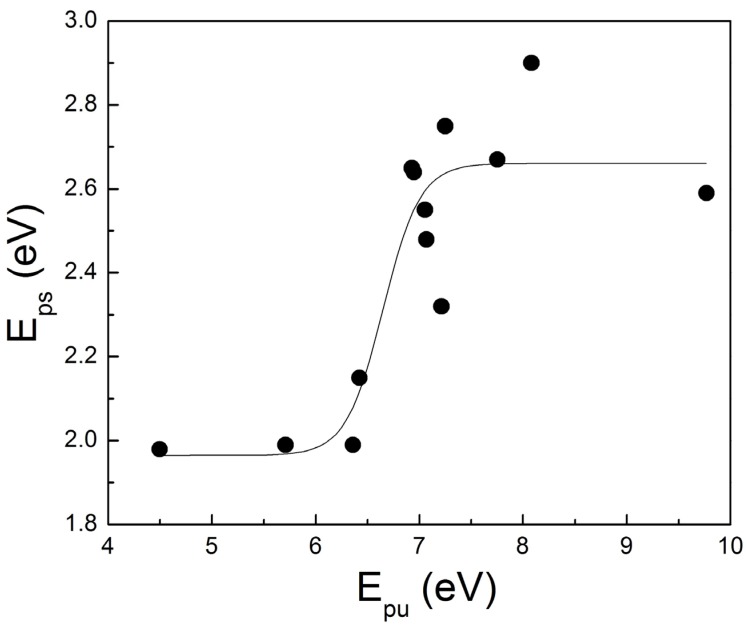
The correlation of screened (*E*_ps_) and unscreened (*E*_pu_) plasma energy for continuous TiN films resulted by the D2L analysis of the various TiN samples reported in Table 2 (the line is a guide to the eye).

Comparing the values of the various parameters as a result of the D2L models, we can conclude the following results:

(1) The maximum MFP acquired for the reported TiN films is less than 50 nm; this value, compared with equivalent values for noble metals, can provide a measure of the size limitations for single-crystalline TiN nanoparticles, which might exhibit LSPR,
(2) *E*_ps_, which is associated with the SPP performance of TiN as we will show below, is associated only with *E*_pu_ (see also Figure 5) and there is no explicit relation of *E*_ps_ with any other D2L parameter. The meaning of this observation is that, despite that the screening of the plasma energy in TiN is due to the existence of the interband transitions, similar to Au, the numerical variations of these interband transitions are not strong enough to affect *E*_ps_.
(3) *E*_pu_, which is associated with the conduction electron density, is above 6.9 eV for stoichiometric and highly conductive TiN; the same films exhibit *E*_ps_ close to 2.65 eV as observed by Logothetidis *et al.* [117].

### 2.3. Potential of TiN for SPP Applications

After the extensive review of the optical properties of TiN thin films, we proceed with the evaluation of the potential of TiN surfaces for applications involving SPP. SPP are electromagnetic excitations propagating across a planar conductor/dielectric interface, as a direct consequence of the coupling between the electromagnetic field of light and the resonant plasma oscillation of the conduction electrons of the conductor. Therefore, the optical and electrical properties of the conductor will have severe effects on the SPP behavior at a conductor-dielectric interface. As we have reported in the previous paragraphs, TiN exhibits a unique tunability of its conductivity offering great tailoring potential for SPP devices. 

In order to evaluate the SPP at interfaces involving TiN as a conductor, we initially consider the TiN/AlN interface. We base this choice upon the structural and chemical compatibility of the conducting *fcc* B1-TiN(111) and the dielectric *hcp* w-AlN(0001) (note that the fundamental gap of AlN is 6.2 eV [146]) surfaces that result in stable and sharp TiN/AlN interfaces [147,148,149,150,151,152,153]. The most characteristic description of the SPP across the interface is via the dispersion relation that correlates the frequency of light ω with the wave vector *k_x_* in the direction of propagation of SPP. The general equation that defines the aforementioned relation is the following [32]:

(13) $$k_{x}=\frac{\text{ω}}{c}\sqrt{\frac{{\tilde{\varepsilon }}_{\text{TiN}}∙{\tilde{\varepsilon }}_{\text{D}}}{{\tilde{\varepsilon }}_{\text{TiN}}+{\tilde{\varepsilon }}_{\text{D}}}}$$

where ${\tilde{\varepsilon }}_{TiN}$ and ${\tilde{\varepsilon }}_{D}$ are the complex dielectric functions of TiN and the dielectric (AlN in our case), respectively, and c is the speed of light in vacuum. Given that the dielectric functions are inherently complex, Equation (13) should be modified to:

(14) $$k_{x}=\frac{\text{ω}}{c}Re\left(\sqrt{\frac{{\tilde{\varepsilon }}_{\text{TiN}}∙{\tilde{\varepsilon }}_{\text{D}}}{{\tilde{\varepsilon }}_{\text{TiN}}+{\tilde{\varepsilon }}_{\text{D}}}}\right)$$

when optical attenuation in the conductor exists. A more convenient way of writing the dispersion relation in order to be more easily compared to the experiments is:

(15) $$\hbar k_{x}c=\hbar \text{ω}∙Re\left(\sqrt{\frac{{\tilde{\varepsilon }}_{\text{TiN}}∙{\tilde{\varepsilon }}_{\text{D}}}{{\tilde{\varepsilon }}_{\text{TiN}}+{\tilde{\varepsilon }}_{\text{D}}}}\right)$$

**Figure 6:**
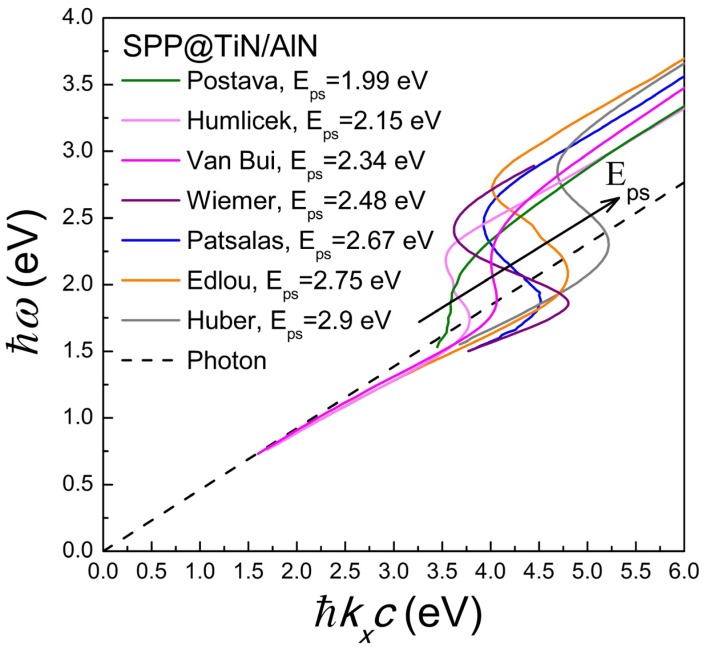
SPP dispersion relations at TiN/AlN interfaces for various qualities of TiN reported by different groups (according to Table 1). The dashed line is the dispersion relation of light in the dielectric.

**Figure 7:**
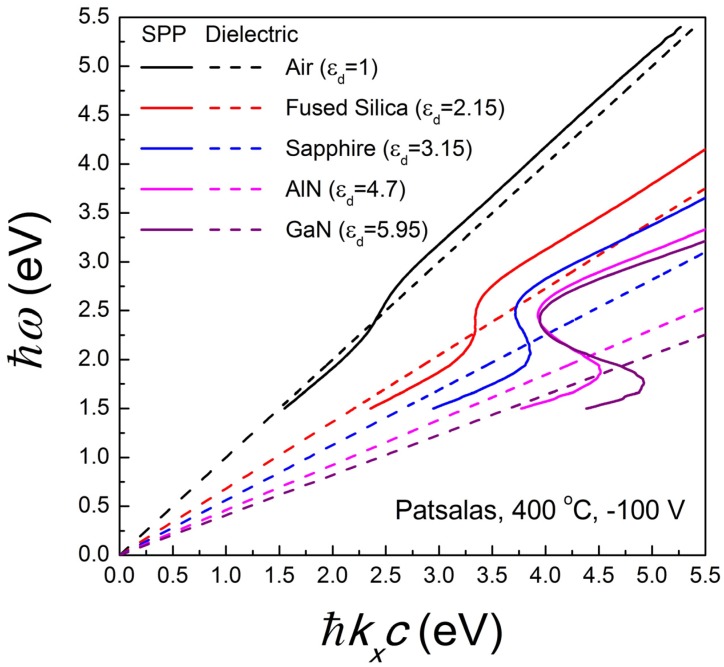
SPP dispersion relations at TiN/Dielectric interfaces for various popular dielectrics and for a highly conductive, low-loss TiN film. The dashed lines are the dispersion relations of light in the various dielectrics.

Figure 6 presents the calculated (via Equation (15)) SPP dispersion relations at the TiN/AlN interfaces using the dielectric function spectra reported by various groups (Table 1) and exhibiting a wide range of *E*_ps_ values. The first significant observation is that in all cases the dispersion relation is characteristic of a lossy metal and getting finite extreme values of *k_x_* [32] due to conduction electron (due to finite Γ_D_ values in Table 2) and dielectric (due to the existence of interband transitions) losses. This has the consequence of the existence of quasi-bound modes at which the slope of the dispersion relation is inversed. Secondly, the characteristic surface Plasmon *E*_sp_ is scaled to the screened plasma energy *E*_ps_ of TiN (*i.e.*, the energy where the actual ε_1_ gets zero value) and not to the unscreened plasma energy *E_pu_*, which is the exclusive characteristic of the conduction electrons. This observation points out the significance of *E*_ps_ not only as a diagnostic tool [117] but also as a critical parameter for the design of plasmonic devices. In addition, Figure 7 demonstrates the SPP dispersion relations at TiN/Dielectric interfaces for various popular dielectrics and for a highly conductive, low-loss TiN film. Air can hardly sustain a SPP mode, while the SPP is stronger and gets bigger extreme *k_x_* values with increasing the dielectric constant of the dielectric material; of special importance is the exceptional SPP performance across the TiN/AlN and TiN/GaN interfaces which are exceptionally stable and sharp [18,147,148,149,150,151,152,153]. Note, however, that SPP-based devices using the TiN/GaN interface are limited to a photon energy range below 3.45 eV, which is the direct fundamental gap of GaN.

### 2.3. Potential of TiN for LSPR Applications

Another field of potential applications of TiN in plasmonics is that of localized surface Plasmon resonance (LSPR) of TiN nanoparticles. The production of TiN nanoparticles was reported [141]. The use of TiN nanoparticles might be beneficial for applications such as solar harvesting and biosensing via Surface-Enhanced Raman Scattering. The optical response of TiN nanoparticle assemblies can be reasonably described by the Maxwell-Garnett Effective Medium Approximation (MG-EMA), which describes the effective optical response of a composite material consisting of a host (matrix) phase and isolated inclusions of the secondary phase [154]:

(16) $$\frac{\tilde{\varepsilon }-{\text{ε}}_{\text{m}}}{\tilde{\varepsilon }+2{\text{ε}}_{\text{m}}}=f_{\text{i}}\frac{{\text{ε}}_{\text{i}}-{\text{ε}}_{\text{m}}}{{\text{ε}}_{\text{i}}+2{\text{ε}}_{\text{m}}}$$

where *f*_i_ is the volume filling ratio of the inclusions, ε*_i_* is the reference dielectric function of the TiN inclusions and ε_m_ is the reference dielectric function of the host (matrix).

**Figure 8:**
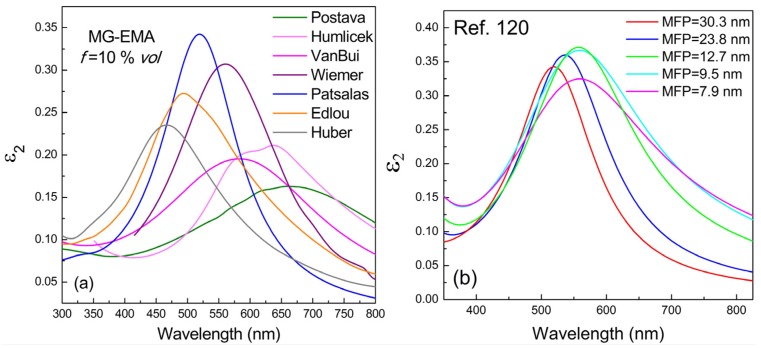
ε_2_ spectra of TiN nanoparticles in air calculated by MG-EMA using TiN dielectric functions retrieved from (**a**) the literature, and (**b**) [120], where a variety of stoichiometric TiN films of varying MFP are reported.

**Figure 9:**
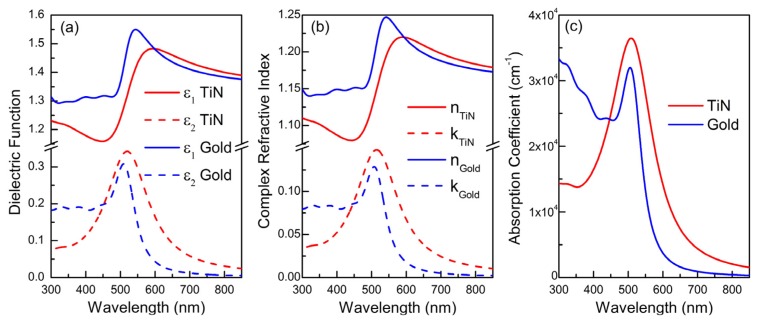
Comparison of the spectra of (**a**) the real ε_1_ and imaginary ε_2_ parts of the dielectric function; (**b**) the refractive index n and the extinction coefficient *k*; and (**c**) the absorption coefficient of nanoparticle assemblies of TiN (from [120]) and 40 nm Gold.

Given that the MFP of conduction electrons of TiN films is less than 250 nm (according to the data presented in Table 2), we used directly the reported dielectric functions of TiN in MG-EMA; we also considered ε_m_ = 1 assuming air as the host medium. The ε_2_ spectra of TiN nanoparticle assemblies in air with filling ratio *f*_TiN_ = 10% *vol* calculated by MG-EMA are presented in Figure 8. In particular, Figure 8a,b present results determined using dielectric functions retrieved from literature and covering a wide range of *E*_ps_ and *E*_pu_, or retrieved from [120], where a variety of stoichiometric TiN films of varying MFP (and consequently grain size) are reported, respectively. In all cases, strong LSPR bands are observed. The narrower and stronger band is located just above 500 nm and resembles the far field LSPR behavior of gold, as it is clearer presented in Figure 9; in particular, Figure 9 shows the spectra of (a) the real ε_1_ and imaginary ε_2_ parts of the dielectric function, (b) the refractive index *n* and the extinction coefficient *k*, which are associated with ε_1_ and ε_2_ via the relations ε_1_ = *n*^2^–*k*^2^ and ε_2_ = 2 *nk*; and (c) the absorption coefficient α = 4 π*k*/λ, of nanoparticle assemblies of TiN (from [120]) and 40 nm Gold. Gold exhibits higher quality factor of LSPR [λ/Δλ (FWHM)] but higher dielectric losses at short wavelengths than TiN.

**Figure 10:**
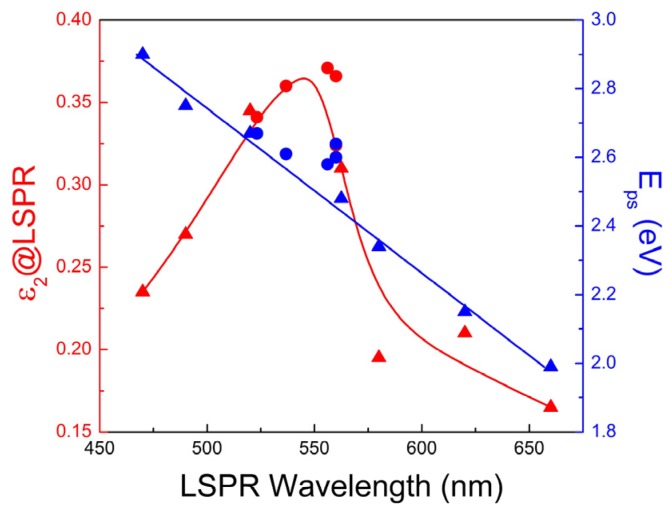
ε_2_ value at LSPR wavelength (red symbols) and the *E*_ps_ parameter (blue symbols) *vs.* the LSPR wavelength. All reported values are deduced by the ε_2_ spectra of TiN nanoparticles in air calculated by MG-EMA using TiN dielectric functions retrieved from the literature (triangles), and [120] (disks) and presented in Figure 8. The lines are guides to the eye.

The spectral variation of the LSPR band of TiN nanoparticles extends to an exceptional range, which covers almost the whole visible range in expense, however, of the strength of the LSPR bands. This observation confirms and supports the great potential and perspectives of TiN plasmonic nanostructures reported recently [155]. The variation of the maximum value of ε_2_ at the LSPR wavelength *vs.* the LSPR wavelength exhibits a distinct maximum above 500 nm (Figure 10, red symbols), in contrast to the monotonous increase of max-ε_2_ with the LSPR wavelength observed for Ag nanoparticles [156]. Finally, the importance of *E*_ps_ is also demonstrated for the case of LSPR, as well. Indeed, the spectral location of LSPR of TiN nanoparticles is straightly scaled with *E*_ps_ as it is demonstrated in Figure 10 (blue symbols). 

## 3. Conclusions

In this work, we reviewed the optical properties of TiN films grown by various techniques and on various substrates as reported by several research groups worldwide. The electronic conductivity of TiN is due to the semi filled *d*-band of Ti. Its optical properties are affected by the co-existence of Ti-*d* conduction (free) electrons and the N-*p* to Ti-*d* interband transitions. Consequently, the dielectric function spectra of TiN can be fitted by a combined model which consists of a Drude term that describes the conduction electrons, and two Lorentz oscillators that describe the interband transitions. The phenomenological parameter *E*_ps_, which has no explicit physical meaning, is associated mostly with the unscreened plasma energy *E*_pu_ of the conduction electrons of TiN and determines the SPP and LSPR characteristics of TiN plasmonic devices. In particular, the SPP performance of TiN/AlN and TiN/GaN planar interfaces is remarkable. In addition, varying the composition and microstructural characteristics of TiN results in variations of *E*_ps_, which in its turn can be used to tailor the spectral position of LSPR emerging from assemblies of TiN nanoparticles. However, the spectral variation of TiN’s LSPR comes at expense of the LSPR strength. The strongest LSPR observed for TiN nanoparticles is located above 500 nm and resembles that of gold
